# Cooperative Object Transport in Multi-Robot Systems: A Review of the State-of-the-Art

**DOI:** 10.3389/frobt.2018.00059

**Published:** 2018-05-25

**Authors:** Elio Tuci, Muhanad H. M. Alkilabi, Otar Akanyeti

**Affiliations:** ^1^The Department of Computer Science, Middlesex University, London, United Kingdom; ^2^The Department of Computer Science, University of Kerbala, Karbala, Iraq; ^3^The Department of Computer Science, Aberystwyth University, Aberystwyth, United Kingdom

**Keywords:** multi-robot systems, cooperative object transport, pushing, pulling, caging

## Abstract

In recent years, there has been a growing interest in designing multi-robot systems (hereafter MRSs) to provide cost effective, fault-tolerant and reliable solutions to a variety of automated applications. Here, we review recent advancements in MRSs specifically designed for cooperative object transport, which requires the members of MRSs to coordinate their actions to transport objects from a starting position to a final destination. To achieve cooperative object transport, a wide range of transport, coordination and control strategies have been proposed. Our goal is to provide a comprehensive summary for this relatively heterogeneous and fast-growing body of scientific literature. While distilling the information, we purposefully avoid using hierarchical dichotomies, which have been traditionally used in the field of MRSs. Instead, we employ a coarse-grain approach by classifying each study based on the transport strategy used; pushing-only, grasping and caging. We identify key design constraints that may be shared among these studies despite considerable differences in their design methods. In the end, we discuss several open challenges and possible directions for future work to improve the performance of the current MRSs. Overall, we hope to increasethe visibility and accessibility of the excellent studies in the field and provide a framework that helps the reader to navigate through them more effectively.

## Introduction

This paper reviews recent research works in MRSs targeting cooperative object transport scenario. A MRS is robotic system consisting of more than one robot (see Cao et al., 1997). MRSs are a promising alternative to automate tasks that are beyond the competency of single robot systems. Transporting big objects, surveillance of vast areas, or robot tasks that can be decomposed into smaller tasks so that they can be carried out simultaneously by several robots are examples of application domains particularly suited for MRSs (Yan et al., 2013). In addition, MRSs, comprised of many but simple individuals, may be cheaper to build and easier to program than a complex robot capable of performing similar tasks (Farinelli et al., 2004; Cai and Yang, 2012; Yan et al., 2013; Khamis et al., 2015; Jiang et al., 2016). MRSs are also potentially more resilient to a large variety of hardware or software failures; when one robot fails or makes a mistake, the others can still complete the task successfully (Parker, 1998).

Although the members of a MRS can be designed or programmed to compete with each other (see Martín H. et al., 2010), the majority of the previous studies have investigated how group members can work together to achieve a common goal (i.e., cooperation). However, so far the scientific community has failed to agree on a formal definition for cooperation. For some authors, it is sufficient to refer a MRS as cooperative as long as its members share a common goal, even if they have zero interaction (Wang et al., 1994; Quinn, 2004). For others, the definition of cooperation is more strict. A MRS is assumed to be cooperative only if the robot task can not be serialised (i.e., single robot can not complete the task in a sequential manner), and specific cooperation mechanisms should be in place so that the robots can coordinate their actions, and possibly complement each others’ capabilities (see Kube et al., 1993; Brown and Jennings, 1995; Cao et al., 1997; Iocchi et al., 2000; Yan et al., 2013). The underlying process that enables cooperative MRSs is generally referred to as coordination of actions (see Kube and Bonabeau, 2000; Simmons et al., 2001; Emery et al., 2002; Farinelli et al., 2004).

Here, for the first time, we provide a comprehensive review on research studies that focus on one application domain; cooperative object transport, the term is coined after (Groß and Dorigo, 2004). Cooperative MRSs are generally employed when the object is too heavy, too large, or has a complex shape so that it can not be transported by a single robot. However, this is not a strict requirement; not all group members need to participate in the physical act of transport; carrying or pushing/pulling the object. Cooperation can still be achieved when a single or few robots transport the object, and the others plan the coordination and navigation of the transporters along a desired trajectory, or clear the way from obstacles (e.g., see Habibi et al., 2015).

Autonomous MRSs capable of cooperative object transport can be extremely effective in a variety of applications that have high economic and societal impact potential; e.g., waste retrieval and disposal, de-mining, or operations requiring object manipulation in environments where direct human intervention is impossible or impractical, such as in space or in deep sea (Huntsberger et al., 2000; Parker and Zhang, 2006; Woern et al., 2006). Thanks to parallelism and decentralised nature of MRSs, the robots apply spatially distributed forces (i.e., pushing, pulling or lifting at different locations) around objects. The physical separation and the independent actions of different agents can potentially generate a group dexterity that a single robot can hardly achieve, irrespective of its sophistication and power (see Brown and Jennings, 1995). This property is particularly important in cooperative transport tasks, where the independent exertion of multiple pushing/pulling forces in different points of an object can allow the group to generate precise translation/rotation manoeuvres in order to avoid obstacles during transport.

Due to its relevance, cooperative transport has been studied in recent years by research works that have extensively looked at different aspects related to the coordination and synchronisation of the forces required to initiate and sustain the transport of objects that can not be transported by a single robot. The research on cooperative transport in MRSs has been progressing by investigating and testing the potentialities of a variety of different methodological approaches, that are generated by integrating, with different modalities, the various available alternatives for what concerns methods and techniques to design the mechanisms underpinning the desired group responses, means for inter-robot communications, transport techniques, evaluation scenarios, etc. The objective of this paper is to review and at the same time to provide a navigation framework to order and critically evaluate this rather heterogeneous and fast growing body of literature. We employ a rather coarse-grain categorisation system that distinguishes and orders the research works with respect to the type of transport strategy used by the group to cooperatively move the object. We believe that this categorisation system represents a helpful perspective to account for the scientific progress made by a methodologically diverse body of literature, and to identify open challenges and promising directions for further work to improve the transport capabilities of MRSs.

We review and categorise the research works using three categories, each of which is discussed in a separate section:

1. Pushing-only strategy (see section 2): robots are not physically attached to the object, and transport is achieved by pushing the object.
2. Grasping strategy (see section 3): robots are physically attached to the object, and transport is achieved by either pushing or pulling (or both) the object.
3. Caging strategy (see section 4): this strategy is similar to the pushing-only strategy. Robots are distributed to entrap the object (i.e., caging) and they hold the object tightly during transport.

We decided to separate pushing-only and caging strategies even though they share some characteristics. This is because the latter is not only concerned with transporting the object but also maintaining an object closure at all times. This additional requirement imposes unique design challenges which influence the communication and coordination strategies employed by the robots (see Hekmatfar et al., 2014). The reader should be aware that cooperative transport has also been studied in MRSs that, due to their characteristics, they do not fit in any of our three categories. In particular, cooperative transport has been studied in a group of aerial robots (Michael et al., 2011; Bernard et al., 2011) required to carry heavy objects using cables. Moreover, cooperative transport has gained significant attention in micro-scale applications where micro-robots (i.e., robots with sub-millimetre or smaller dimensions capable of manipulating micro-objects including living cells) have been designed to perform micro-manipulation and micro-assembly tasks such as molecular delivery to targeted cells, minimally invasive surgeries, tissue engineering, and other general micro-manipulation applications (Hu et al., 2011; Shahrokhi and Becker, 2016; Rahman et al., 2017). We decided to exclude from this review these and other similar research works based on transport strategies alternative to the pushing, grasping, and caging strategy described above.

In section 5, we provide an informative and constructive discussion on the state of the art of MRSs engaged in cooperative transport that helps to identify objectives for interesting future directions of research. Contrary to other similar review papers, we do not employ the classic and frequently used dichotomous view that distinguishes MRSs in those controlled in a centralised and those controlled in a decentralised way (see Cao et al., 1997; Bahçeci et al., 2003; Bayindir and Şahin, 2007). We believe that, in the context of cooperative transport, the use of such a dichotomous perspective would blur important methodological details that largely contribute to the identity and the originality of every single study. We rather complement the review framework based on the type of transport strategy illustrated above, with references to the eventual presence of any *key element* in the MRS’s architecture, and we comment on the type of communication used to achieve the coordination of action among the group members. In our view, the *key element* can be either a member of the group or an element external to the group (e.g., a server), that orchestrates the dynamics of the group by regulating some or the totality of the actions of those agents that are subordinated to its decisions. When the *key element* is internal to the group, it is generally represented by an agent that is either structurally or functionally different from the other robots of the group (e.g., a leader). When the competencies and contributions of the *key element* to the group performance can not be dynamically allocated and more importantly re-allocated to any of the other members of the group, the *key element* undoubtedly represents a single-point of failure of the system. This is because a failure of the *key element* inevitably leads to the failure of the entire system.

Before we start, we would like to clarify few points. First, our review mainly focuses on research studies that use mobile robots. These robots typically vary in body length, and methods for locomotion (e.g., legged or wheeled robots). The studies using other types of robots (e.g., aerial and aquatic robots, and micro/macro robotic manipulators) are omitted unless there is a specific point to be made. Second, even within the mobile robotics literature, there is a large body of work, which is impossible to cover in one review paper. Hence, we try to select the most representative studies that employ different transport, coordination and control strategies. Third, we define four terms that help us to better describe various MRSs approaches: MRSs that use direct or indirect communication, and homogeneous and heterogeneous MRSs. In direct communication, the members of a MRS send/receive messages to/from each other using a dedicated communication network. Messages are often transmitted via text, sound or light using wireless communication protocols. Based on these protocols, message exchange can be private (i.e., between two or selected group members), local (i.e., among neighbours within close proximity) or global (among all members). In indirect communication, the robots are not allowed to communicate with each other explicitly. Instead, they communicate implicitly using the object they transport and/or through the changes in the environment they operate in. In homogeneous MRSs, all group members are identical with same hardware (i.e., physical) and software (i.e., functional) designs, whereas in heterogeneous MRSs, at least one group member is physically and/or functionally different from the others. Homogeneous groups are more frequently found in swarm robotics, a sub-field of the MRSs research area where the robots mimic main characteristics and behaviour of social insects, such as ants and bees (see Şahin, 2005). The structural and functional homogeneity of a robotic swarm is inspired to the genetic similarities of “workers” in social insects. The group homogeneity is supposed to make the group more scalable with respect to its size and more resilient to individual failure, since in principle any robot can replace any other identical member of the group. The group homogeneity does not preclude the possibility that a certain amount of functional diversification could characterise the group members, as long as any behavioural specialisation emerges during the life of the group, and it is in principle reversible. Cooperative object transport scenarios often require complex and diversified behavioural competencies that scientists have very frequently implemented by exploiting structurally and/or functionally heterogeneous rather than homogeneous groups. Advantages and drawbacks of the use of heterogeneous groups in the context of cooperative transport will be further discuss in section 5.

## The Pushing-Only Strategies

Pushing-only strategies are methods of collectively transporting items by exerting pushing forces on the item. These type of strategies are primarily employed by robots that can not pull objects, since they have no means to grasp them. Pushing-only strategies may appear to be relatively simple methods of cooperative transport. However, on top of the challenges common to all transport strategies (e.g., the alignment of forces required to initiate the transport, etc.), pushing-only strategies require a significant amount of coordination of actions to sustain the transport. The item may move on a very inefficient trajectory unless the robots carefully manage frictional, gravitational, and dynamical forces to stabilise the direction of transport. Table 1 summaries the main characteristics of the research works reviewed in this section. Generally speaking, it is worth noticing that the large majority of these works are based on homogeneous groups, where the robots’ controller is designed using a behaviour-based methodology (see Brambilla et al., 2013, for further details). Groups exploiting indirect communication prevail on groups exploiting forms of direct communication. Half of the studies look at a simplified transport scenarios, where the problems related to the initial alignment of pushing forces is solved by initialising the robots very close to the object, facing the same side of the object (see no random initial positions in Table 1). In the following, we review these works, by emphasising objectives and achievements.

**Table 1:** Main characteristics of research works using a pushing-only transport strategy.

	Experimental setting						Robustness Test		Control and communication			Similar studies
	nr. robots	group type: -homogeneous (ho) -heterogeneous (he)	env. with obstacles	obj. shape	transport direction	random initial positions	obj. mass	obj. dim	controller design method	autonomous allocation of roles	communication	
Kube et al., 1993	5	ho	no	rectangular prism	arbitrary	no	no	no	behaviour based	n/a	indirect	Kube and Zhang, 1997; Kube and Bonabeau, 2000
Mataric et al., 1995	2	ho	no	rectangular prism	towards a goal	no	no	no	behaviour based	n/a	direct	
Gerkey and Matarić (2002)	3	he	no	rectangular prism	towards a goal	no	no	no	other	Yes	direct	
Yamada and Saito, 2001	4	ho	no	rectangular prism	towards a goal	no	no	no	behaviour based	n/a	indirect	
Jianing Chen et al., 2015	20	ho	no	cylinder, rectangular, triangular prism	towards a goal	yes	yes	no	behaviour based	n/a	indirect	Kapellmann-Zafra et al., 2016
Sugie et al., 1995	4	ho	no	rectangular prism	towards a goal	yes	no	no	other	n/a	direct	
Wang and de Silva, 2006a	2	he	yes	rectangular prism	towards a goal	yes	no	no	automatic	no	direct	
Alkilabi et al., 2017	2 to 6	ho	no	rectangular prism	arbitrary	yes	yes	yes	automatic	n/a	indirect	Alkilabi et al., 2016
Fujisawa et al., 2013	4 to 10	ho	no	cylinder	towards a goal	yes	no	no	behaviour based	n/a	indirect	
Neumann et al., 2014	2	ho	no	rectangular prism	predefined path	yes	no	no	other	n/a	indirect	
Tang and Parker, 2005	2 to 3	he	no	rectangular prism	arbitrary	Yes	no	no	behaviour based	no	direct	
Parker, 2000	2	he	no	rectangular prism	arbitrary	no	no	no	other	no	direct	
Wang and de Silva, 2006b	3	ho	yes	rectangular prism	towards a goal	no	no	no	automatic	n/a	indirect	Sen et al., 1994

A transport direction is classified as “arbitrary” when the robots autonomously choose where to transport the object. The “no random initial positions” category refers to specific initial spatial arrangements of the robots relative to the object that tend to facilitate the alignment of pushing forces. Robustness tests with respect to the object mass and dimensions are tests in which the effectiveness of group transport strategies are evaluated with objects of different mass, and different size. The classification of the controller design methods is the one discussed in (Brambilla et al., 2013).

The study in (Kube et al., 1993) can be considered the pioneering work targeting a cooperative transport task by a homogeneous group of simple robots that can only push the object (i.e., a box). This study is considered to be the first research work that formally represented in “hardware” the dynamics of cooperative transport. The authors demonstrate that coordinated efforts in a box pushing task are possible without the use of direct communication or robot differentiation. The group exploits the physical interactions among the robots and between the robots and the object to initiate and to sustain the transport. In (Kube and Zhang, 1997; Kube and Bonabeau, 2000), the authors further develop the model described in (Kube et al., 1993) with the addition of a stagnation recovery strategy. Stagnation refers to a deadlock condition in which the robots cancel each others’ pushing forces due to the way in which they are positioned around the object. The authors also evaluate the group transport strategies with objects of different shapes in scenarios in which the objects have to be transported towards a moving target.

Mataric et al. (1995) propose the use of direct communication to improve the coordination of a homogeneous group of two six-legged robots required to cooperatively transport a rectangular box toward a target. Published during a time of disaffection for the classic AI paradigm, this study aims to demonstrate that tasks requiring complex coordination of actions among physical robots can be successfully accomplished without the robots having any model of the world and without being able to make any predictions on the consequences of their actions. Robots’ controller is designed using a behaviour based methodology (Brooks, 1986), and communication is used by the agents to exchange their sensors readings and to implement a turn-taking protocol. To facilitate the initial alignment of pushing forces, the robots are positioned on the left and on the right end of one of the longest object’s side. The results indicate that the use of communication and of the turn-taking protocol significantly helps the robots to improve the overall group performance.

Gerkey and Matarić (2002) illustrate the performances of a group of three robots in which one element of the group plays the role of the watcher, and the other two robots play the role of the pusher. The watcher perceives the object and the goal destinations, and its main duty is to lead the team by providing the other robots information concerning the direction of transport. The pushers push the cuboid object without perceiving the goal destination which remains hidden behind the box that occludes their view. The robots rely on a direct form of communication for the coordination of their actions. The transport trajectory is free from obstacles, and roles are assigned using an auction-based system (i.e., MURDOCH architecture, see Gerkey and Matarić, 2001). The heterogeneous group manages to successfully transport the object in straight and curved trajectories. The system also proved to be resilient to the failure of one of the pusher, and to a certain extent to the failure of the communication mechanism underpinning the watcher-pusher interaction. However, the system heavily relies on the capabilities of the single watcher, which acts as a *key element* that gathers sensory information sent by the pushers and generate the group response by instructing the pushers on how to move.

The study illustrated in (Yamada and Saito, 2001) is also conceived in support of a theoretical perspective alternative to the classic AI, since its main goal is to demonstrate with physical robot experiments that an environment selection task and a cooperative box-pushing task can be both carried out by a homogeneous group of robots where agents are guided by a reactive controller. Contrary to (Mataric et al., 1995) and (Gerkey and Matarić, 2002) which advocate for the use of direct communication, the results illustrated in (Yamada and Saito, 2001) demonstrate that indirect communication is sufficient to cooperatively transport an object toward a target area. The robots can operate in a simple environment where individual robots are required to push light boxes, or in complex environments where multiple robots are required to cooperatively push a heavy box. The mechanisms underlying the environment selection task operate under the assumption that there is no moving object except the robots. Moreover, it is assumed that during pushing, no wheels slippage is experienced by the robots even in those cases in which the object does not move when subject to pushing forces. These assumptions are required to allow the robots to discriminate between those cases in which the box is light enough to be transported individually, from those cases in which the box is so heavy to require a cooperative response.

Jianing Chen et al. (2015) propose an alternative group transport method which exploits occlusion, rather than trying to overcome the limitations imposed by it. The robots are designed to push the object across the portion of its surface, where it occludes the direct line of sight to the goal. In this study, a group of twenty e-puck robots (see Mondada et al., 2009) are required to transport a cylindrical object towards a goal. The robots push the object only when they can not see the goal destination. This simple behaviour results in transporting the object towards the goal without using any form of direct communication. The authors also provide an analytical proof of the effectiveness of the method, and results of successful empirical tests with a cuboid and a triangular objects are discussed. In (Kapellmann-Zafra et al., 2016), the occlusion-based strategy discussed in (Jianing Chen et al., 2015) is tested in a task in which the robots are required to transport an object towards a moving target, represented by another robot.

The study described in (Sugie et al., 1995) is one of the first to address the problem of designing push-only strategies in a dynamic environment that incorporates obstacles. The authors describe a system in which the robots infer other robots’ intentions by observing their behaviour and cooperate based on those inferred intentions. A camera placed on the ceiling of the robots arena communicate to each robot the position of all other robots, obstacles, boxes to be transported, and final destinationsof each box. An algorithm made of a task planner, a pushing action planner, and a dynamic obstacle avoidance function guides the robots during the task execution. In this as in other similar studies in which the control algorithm relies on a global view of the environment, the group transport strategy, although particularly effective to manoeuvre the object in a complex environment with obstacles, would not tolerate a fault to its multiples *key elements*, such as the camera and the task planner.

Wang and de Silva (2006a) consider a heterogeneous group of robots that is required to cooperatively transport a box by removing obstacles that abstract the way to the final transport destination. The authors propose an approach based on the use of a force/motion control system. Three different types of agent are used in this approach: a vision agent that has a global view of the environment to generate positions and orientation coordinates of all robots, the object, and the obstacles; a learning agent responsible for generating cooperation plans based on an optimisation approach that integrates reinforcement learning and genetic algorithm; two physical robots that execute the plan generated by the learning agents. The plan may require one robot to leave the transport to remove obstacle/s obstructing the way to the final transport destination. The study demonstrates the feasibility and the effectiveness of the proposed method using experiments with two small prototype robots. Both the vision and the learning agents are *key elements* whose contribution is vital for the correct functioning of the MRS.

Alkilabi et al. (2017) demonstrate that effective coordination of actions for initiating and sustaining the transport of heavy objects to be moved in an arbitrary direction can be obtained by homogeneous groups of robots by exploiting a relatively simple form of indirect communication based only on the possibility to perceive the movements of the object. In this study, physical e-puck robots are equipped with an optic-flow sensor whose readings are used to distinguish between cases in which the robots pushing forces contribute to moving the object from those cases in which the robots efforts do not result in any significant object movement. The possibility to discriminate between the above mentioned two circumstances is vital for the initial alignment of pushing forces and for sustaining the transport. The authors show that the transport strategies are scalable with respect to the group size, and robust enough to deal with boxes of various mass and size. In a complementary study illustrated in (Alkilabi et al., 2016), the authors complement the robots’ neuro-controller, initially designed to support the object transport in an arbitrary direction, with mechanisms to direct the transport towards a specific target location.

A cooperative transport study that uses indirect communication via artificial pheromone is described in (Fujisawa et al., 2013). In this study, a group of ten robots can sense and lay on the terrain a volatile alcohol substance that mimics the effect of ants’ pheromone during trail formation. The task requires the robots to perform a random search to find a food item (i.e., heavy object), and to transport it to a goal location (i.e., the nest). The pheromone-based communication is used by the robots to recruit other nest-mates when a food location is identified. The results indicate that pheromone-based communication contributes to reducing the task completion time, in comparison to the case in which the robots depend completely on a random walk to congregate at the food. The study also shows that the pheromone-based communication is effective only with a relatively small number of robots in the environment. When a larger group of robots is used, the pheromone-based communication has less impact on the completion time, as many robots are likely to find the food and begin the cooperative transport before the trail is formed.

In (Neumann et al., 2014), an algorithm running on an external server controls a group of robots required to push a box on straight and circular trajectories defined by the experimenter. The algorithm generates informations concerning where the robots have to apply pushing forces and the magnitude of the forces needed to transport the box. Position and orientation of the robots and of the box are measured using an ultra-wide band tags placed on the robots as well as the box. The readings generated by the force sensors and data relative to the robots’ position generated by the ultra-wide band system are routed to a central server, which in turn calculates the robots’ required speed and sends the commands to the robots accordingly. The robots execute the commands to generate the desired forces and torques on the object in order to move it along a planned trajectory. The study demonstrates the validity of the proposed method using two Pioneer robots equipped with hinged force sensors extension. The server running the control algorithm is the *key element* which manages the robots’ actions by sending instructions to each robot using a direct form of communication, supported by a wireless communication network. Such type of direct communication tends to suffer from scalability issue, since the communication load increases when the number of robots increases. This may cause a decrease in system performance or in extreme cases, it can result in an overall system failure. Moreover, the scalability of the transport strategies may be also hindered by issues related to the design of network topologies and to the communications protocols (see Cao et al., 1997).

The cooperative object transport scenario using a pushing only strategy has also been used in various research studies as a benchmark task to evaluate the functional characteristics of various control policies (see Sen et al., 1994; Parker, 2000; Tang and Parker, 2005; Wang and de Silva, 2006b).

## The Grasping Strategies

Grasping strategies are methods by which the robots physically attach to an item to be able to collectively transport it. Thus, grasping strategies can only be exploited by robots which possess the mechanisms to grasp an object. There exists a variety of mechanisms that allows a robot to physically connect to an object, some of which allow the robots not only to grasp but also to lift an item. Compared to pushing-only strategies, grasping strategies provide a better control over the transported object, since once grasped, the object can be either pushed or pulled. However, stable and effective grasping strategies often require the robots to optimally distribute around an object in order to avoid undesired effects, such as the object touching the ground, or the load being distributed in an unbalanced way among the robots. To avoid the challenges related to the effective positioning of the robots around the object, the majority of the research works reported in the literature focus on the development of grasping strategies by groups of robots that are pre-attached to the object and optimally positioned around it before starting the transport (see also Table 2). The work described in (see Sasaki et al., 1995) is one of the few in which the authors develop an algorithm to allow a homogeneous group of robots to find the optimal arrangement around an object that has to be lifted and transported to a final destination. In this study, the robots know the shape of the object. They estimate the object mass and mass centre position by lifting the object, and they use these estimates to optimally distribute the grasping points around the object.

**Table 2:** Main characteristics of research works using a grasping transport strategy.

	Experimental setting							Control and communication		Similar studies
	nr. robots	group type: -homogeneous (ho) -heterogeneous (he)	autonomous allocation of roles	env. with obstacles	obj. shape	transport direction	pre-attached	controller design method	communication	
Sasaki et al., 1995	3	ho	n/a	no	irregular convex shaped	object only lifted, not transported	no	other	direct	
Kosuge and Oosumi, 1996	2	he	no	no	rectangular prim	arbitrary	yes	other	direct	Kosuge et al., 1998; Takeda et al., 2002
Wang and Schwager, 2016	4	he	no	yes	rectangular prism	know to leader	yes	other	indirect	
Wang et al., 2016	4	he	no	yes	square prism	known to leader	yes	other	indirect	
Farivarnejad et al., 2016	4	ho	n/a	no	rectangular prim	predefined path known to all robots	yes	other	indirect	
Habibi et al., 2015	5 to 7	he	no	yes	irregularconvexshaped	predefined path known to guide robots	yes	other	direct	Habibi et al., 2014
Yufka and Ozkan, 2015	2–3	ho	n/a	no	rectangular prism	towards a goal known to all robots	yes	other	direct	
Tuci et al., 2006	6	ho	n/a	no	cylinder	towards a goal visible by all robots	no	automatic	indirect	Groß and Dorigo, 2008; Gross and Dorigo, 2009
Ferrante et al., 2010	3–4 (sim)	ho	n/a	yes	cylinder	towards a goal known to some robots	yes	behaviour based	direct	Campo et al., 2006
Berman et al., 2011	4–10 (sim)	ho	/ao	no	cylinder	towards a goal known to all robots	no	behaviour based	indirect	Wilson et al., 2014; Gelblum et al., 2016; Guo et al., 2017
Stilwell and Bay, 1993	11 (sim)	he	no	no	object on pallet	towards a goal known to all robots	yes	other	direct	Johnson and Bay, 1995; Bay, 1995; Pereira et al., 2002; Loh and Traechtler, 2012
Hichri et al., 2016	3 (sim)	ho	n/a	no	rectangular prism	towards a goal known to all robots	no	other	direct	Wang et al., 1994; Yamashita et al., 2003
Stroupe et al., 2005	3	he	no	no	rectangular prism	towards a goal known to the robot leader	no	behaviour based	indirect	Hashimoto et al., 1993
Machado et al., 2016	2	he	no	yes	rectangular prism	towards a goal known to the robot leader	yes	behaviour based	indirect	Soares et al., 2007

“pre-attached” refers to an initial configuration in which the robots are already attached to the object. See also caption Table 1 for further illustration of the descriptive categories.

Most of the research works on cooperative transport using grasping strategies rely on the presence of a robot leader to generate the desired motion trajectory of the object. In these studies, no mechanisms for a dynamic allocation of roles are contemplated. Thus, the leader can be considered a *key element* which, if it fails, the entire group stops working. A leader/follower approach is described in (Kosuge and Oosumi, 1996), where a group of two robots cooperatively transport a long cuboid object pre-attached to them using free rotational joints. The control algorithm requires the presence of a leader robot that is in charge of implementing a specific motion trajectory. The follower supports the leader in the transport of the object along the desired trajectory by coordinating its actions through the perception of the forces applied to the object. In (Kosuge et al., 1998), the authors extend this algorithm originally designed and tested on holonomic robots with velocity-controlled actuators to nonholonomic mobile robots driven by two wheels. In (Takeda et al., 2002), the authors further improve the control algorithm by adding a collision avoidance unit to enable the robots to transport a single object in more complex environments with obstacles.

A leader/follower approach is also exploited in (Wang and Schwager, 2016) and in (Wang et al., 2016). Wang and Schwager (2016) describe a kinematic controller for a group of four robots, in which the robot leader pulls the object and defines the directionof transport, and the robots follower push the object to sustain the leader effort. The model requires the robots to have information beforehand of the friction coefficients, the mass of the object, and the total number of the robots forming the group, in order to measure the velocity and acceleration at the centre of mass of the object. The robots are manually attached to the object with a fixed connection established by a one DOF gripper. Three experimental set-ups are studied with different types of leaders (i.e., an autonomous robot, a robot teleoperated by a human, and a human leader), while the characteristics of the robots follower are kept unchanged in all three experimental set-ups. The results of the study demonstrate the followers’ ability to effectively coordinate with all types of leader by following the leader-defined direction of object motion. In (Wang et al., 2016), the kinematic control described in (Wang and Schwager, 2016) is extended in order to allow a group of four custom-built omnidirectional robots (i.e., OuijaBots) to transport a longitudinal object along trajectories requiring the object to be rotated in order to cross a narrow corridor.

The objective of the study described in (Farivarnejad et al., 2016) is to design controllers that drive a homogeneous group of four “Pheeno” robots (see Wilson et al., 2016) to collectively transport a rectangular load at a desired speed along a straight path in a target direction. No distinction in leader/follower is assumed. Moreover, the robots do not have global localisation or communication capabilities, and they lack information about the payload dynamics, the number of robots in the transport team, their distribution around the payload, and the layout of the environment. It is assumed that each robot can measure its speed and heading, and it is given access to the desired target direction of the transport. The position and orientation of the robots with respect to the object are also known since all robots are rigidly pre-attached to the object. Each robot is equipped with wheel encoders to estimate its velocity and a compass to calculate its heading. The results demonstrate the robots’ capabilities in transporting the object in relatively straight trajectories parallel to the desired path with some drift caused by the noise in the compass measurements and the errors in the odometry due to the wheel slippage.

In (Machado et al., 2016) and in (Soares et al., 2007) robots are controlled with a dynamic control architecture that uses the attractor dynamic approach to behaviour-based robotics (see Bicho and Schöner, 1997). In the most recent work (see Machado et al., 2016), the authors test the control architecture on a group of two physical robots jointly transporting a rectangular prism carried on a payload support base capable of returning bearing and displacement of the load with respect to the robots centre of mass. The leader robot, equipped with an omnidirectional camera, generates the transport trajectories in order to avoid static and moving obstacles that obstruct the transport. The results of the study show that the dynamic control architecture allows the heterogeneous group of mobile robots to operate in complex cluttered environments and to successfully transport loads of different with and length.

Habibi et al. (2014) describe a distributed path planning algorithm that allows the robots to construct a configuration space of the environment in a distributed fashion. A shortest-path tree is constructed using a variation of the Bellman-Ford algorithm (see Bang-Jensen and Gutin, 2008). The algorithm can cope with dynamic obstacles and changes in robot population. The algorithm is successfully tested in simulation and also with a homogeneous group of physical robots (see Habibi et al., 2015) pre-attached to an irregular object. This approach requires some robots to perform the transport while others to map the environment in order to guide the transporting robots in the direction of the goal while avoiding obstacles. While this approach is effective in selecting optimal transport trajectories, it requires the majority of robots to map the environment rather than performing the actual transport task. Yufka and Ozkan (2015) illustrate another motion planning algorithm for a group of homogeneous robots required to transport a heavy object to its final destination. This algorithm requires the robots to know their position in the environment, and also assumes that the robots can directly communicate with each other. Initially, the object trajectory is generated, and then each robot generates its trajectory to satisfy the current formation constraints. The algorithm is successfully tested with groups made of a different number of Pioneer robots pre-attached to the object.

A series of studies published in (Tuci et al., 2006; Groß and Dorigo, 2008; Gross and Dorigo, 2009) looked at the design of neuro-controllers synthesised using evolutionary computation techniques to control homogeneous groups of robots that are not required to be pre-attached to the object to be transported. The robots can physically connect both to each other and to the object. The task requires the robots to transport the object using a gripper mounted on a horizontal active axis that can be used to graspand lift objects (Mondada et al., 2004). The robots can also change the relative orientation of the wheels with respect to the grasping point by rotating their upper body (i.e., the turret with the gripper) with respect to the chassis where the wheels are mounted. The results of these studies demonstrate that the combination of feedback generated by force sensors, the rotating turret mechanism for the effective alignment of pushing/pulling forces, and the possibility to have robot-robot connections generate an extremely effective solution to transport objects of different shapes and sizes towards a static and a moving target without the strong requirement of the robots being pre-attached to the object. In (Campo et al., 2006) and in (Ferrante et al., 2010), the collective transport strategy above mentioned has been exploited to develop two different algorithms for negotiating a common direction of transport by robots carrying an object toward a goal destination in an environment with and without obstacles.

Berman et al. (2011) try to mimic the behaviour of ants during group transport by looking for the individual rules that generate robust group-level responses. The authors observe a particular species of ant (i.e., *Aphaenogaster cockerelli*) in order to extract and reproduce in a simulated robotic system those rules that govern the ants individual actions during a foraging task requiring group transport. Individual rules are validated by comparing the behaviour of simulated and real ants. Other recent studies that follow a similar approach can be found in (Wilson et al., 2014; Gelblum et al., 2016; Guo et al., 2017).

In the remaining of this section, we review a series of studies in which the robots cooperatively transport an object on top of their bodies. Although the robots do not have any means to directly grasp the object, we consider their transport strategies as a type of grasping strategy since the robots align their forces and sustain the transport without losing physical contact with the object as in almost the totality of the works in which robots use grasping devices to physically attach to the object to betransported.

Stilwell and Bay (1993) and Johnson and Bay (1995) describe a MRS designed to collectively transport a single palletised load. A group of simulated “ant-like” robots initially lift and then transport an item by carrying it on top of their bodies. The robots do not require an a priori knowledge about pallet mass, pallet inertia, number of the robots in the group, and their positions relative to the pallet centre of gravity. Coordination of action is achieved by sensing the forces applied to the object during the transport of the rigid pallet. In order to facilitate the dynamics of force distribution across the load, the study proposes a “reactive caster” approach that follows principles similar to those of the passive caster wheels when it aligns itself with the direction of travel. The followers align themselves with the leader by sensing the reaction force applied to their top surface with the force exerted by the leader. In (Bay, 1995), the reactive caster approach is successfully evaluated on physical robots. Any robot of the group can potentially play the role of the leader or of the follower. However, no mechanisms for a dynamic allocation of the role are contemplated. Similar studies in which a pre-selected leader manages the motion trajectory of the object and the coordination of actions is achieved through the physical interaction with the object are presented in (Pereira et al., 2002; Loh and Traechtler, 2012).

Hichri et al. (2016) propose a control algorithm in which an external server globally communicates with the robots to perform the transport task. In this study, a homogeneous group of robots is equipped with manipulators for grasping and lifting an object in order to place it on top of their bodies. Optimal positions for the robots to ensure the stability of the object are calculated based on a a priori knowledge of the number of robots in the group, object’s shape, mass and centre of gravity. The server communicates position information to the robots to approach the object, to lift it, and to carry it to a destination. While carrying the object, the robots keep the desired position relative to the object, thanks to the global knowledge of the environment provided by the external server guiding the robots during transportation. The proposed approach is validated in simulation. The results of this study point to the ability of robots to maintain the stability of the object during lifting and carrying tasks. A similar approach based on the use of an external server to coordinate the robot actions is described in (Wang et al., 1994; Yamashita et al., 2003).

In (Stroupe et al., 2005) the strategy of carrying objects on robots’ bodies is used in combination with a leader-follower approach. The study demonstrates, using physical robots, the capability of grasping, lifting, transporting and positioning objects in a construction task. A group of two rovers are required to manipulate objects in order to build a simple structure in a lunar-like environment. The robots communicate with each other to synchronise the grasping, lifting and placing of the objects in building the structure. The robots coordinate their actions by feeling the forces applied on the carried object using a force-torque sensor located on their manipulators. The followers coordinate with the leader by adjusting their velocity based on force-torque feedback such that the torques and forces on the manipulator remain within the experimentally defined threshold. The results indicate that the team successfully completes the construction task with a low failure rate. Another similar leader-follower cooperative transport study using a direct instead of an indirect form of communication can be found in (Hashimoto et al., 1993).

## The Caging Strategy

Cooperative transport by caging is a special case of the previously discussed pushing-only strategy whereby robots intentionally entrap the object to ensure the object follows the group movements. In the caging strategy, robots arrange themselves around the object in order to form a “closure” that traps the object (Rimon and Blake, 1996). The closure must be maintained during transport to ensure the object does not escape from the robots’ cage. In cooperative transport based on a caging strategy, the object’s shape and size are particularly important features since they bear upon the minimum number of robots required to surround the object.

The simplest form of caging strategy with a small number of robots can be found in (Wang et al., 2004b). This study describes a variable internal force control algorithm to guide a group of three omnidirectional robots required to transport a cuboid object. The robots are cube-shaped therefore they touch the object by a line segment (rather than a point). Only the leader pushes the object while the followers hold the sides of the object tightly, such that no change occurs in the relative position and orientation between the object and each follower robot. The robots coordination is achieved by simply sensing the resultant force applied to the object and its movement. This form of indirect communication through the object is sufficient to allow the followers to maintain the formation and to contribute to the transport by exerting forces to move the object along the trajectory known only to the leader. The main limitation of this study is that the system can not follow an arbitrary trajectory that incorporates sharp turns especially when the velocity is low. Similar examples of the use of a caging strategy with the leader-follower approach can be found in (Wang et al., 2003; Wang et al., 2004a).

Brown and Jennings (1995) propose a *pusher-steerer* approach to cooperative transport which is similar to the one discussed in (Wang et al., 2004b) but without the requirement of maintaining tight contact with the object during transport. The steerer is programmed to follow a predefined path while the pusher exerts the necessary forces to transport the object. The object is placed between the pusher and the steerer. During the transport, the steerer senses the arc length travelled and adjusts its heading to follow the programmed trajectory. Using indirect communication, the pusher follows the change in the object’s configuration by maintaining a fixed orientation relative to the rear face of the object. This approach is similar to a rear-wheel-drive vehicle but implemented with two separate pieces (i.e., the pusher and the steerer). The approach is validated through experiments using two physical robots to transport boxes of varying size and mass along different paths. The results indicate that the robots can successfully maintain the caging while following the programmed trajectory.

Spletzer et al. (2001) describe a cooperative transport task in which caging strategy is achieved using vision to estimate distances and relative orientations of the robots. In this study, a leader robot and two followers are required to transport an object to a destination known only to the leader. Followers maintain a desired distance and relative bearing to the leader in order to form a closure that cages the object. This approach is similar to the “pusher-watcher” approach described in (Gerkey and Matarić, 2002) and reviewed in section 2. Contrary to (Gerkey and Matarić, 2002), in this study, the watcher robot contributes to the transport by caging the object with the pushers. The main drawback of this method is that all robots need to maintain visual contact with each other.

Pereira et al. (2004) propose an algorithm for collective transport using a caging strategy that relies only on the robots’ ability to estimate the object’s orientation and the positions of their neighbours. In this study, three holonomic car-like robots are required to move in the direction of a goal while maintaining a formation trapping a triangular object. Each robot is equipped with an omnidirectional camera to estimate the object orientation and its position with respect to its neighbouring robots. This information is explicitly communicated between the robots to complement their partial knowledge about the object orientation and the position of other robots. The control algorithm assumes that each robot has an imaginary copy of the object attached to it at the object’s origin (i.e., one of the object’s corner). The intersection of these imaginary objects forms a region referred to as the *closure configuration space*. If the origin of the actual object falls inside the closure configuration space, then an object’s closure is accomplished otherwise, the robots have to adjust their positions to satisfy this condition.

In a more complex scenario, (Fink et al., 2007) propose a caging strategy for a group of robots required to transport an L-shaped object on a predefined trajectory. In this study, the robots locally estimate the object closure based on direct communication regarding their position with respect to the object. Controlled by a subsumption architecture, the robots switch from the approach behaviour to the surround behaviour when they are close to the object. In surround behaviour, the robots distribute themselves around the object in order to form the potential caging. This approach requires the robot to know the object’s minimum diameter (i.e., the smallest gap through which the object can fit), maximum diameter (i.e., the maximum distance between anytwo points in the object), and the radius of the caging circle. The robots communicate their states to neighbours until a quorum is reached—that is, when enough robots surround the object, and all are ready to initiate the transport behaviour. During transport behaviour, every robot adjusts its speed depending on the positions of neighbours and the desired trajectory of the object. If for any reason the closure is lost during transport, every robot returns to the surrounding behaviour to resume the transport. The study verifies the effectiveness of the approach using eight differential drive robots equipped with wheel encoders and laser range finders. The results of this study show the stability of the proposed caging strategy in a scenario in which the robots successfully form closures that surround the object while pushing it from an initial position to its final destination. Later in (Fink et al., 2008), the authors extend this approach to allow the robots to operate in a more complex environment that incorporates obstacles. Another study involving a similar caging strategy generated by a subsumption architecture is described in (Eoh et al., 2014).

In (Dai et al., 2016), a control architecture based a fuzzy control methods integrated with the sliding mode method is used to control a heterogeneous group of three physical robots required to collectively transport, using a caging strategy, a convex polygon along different predefined trajectories. The robots have some predefined knowledge about the object shape. Moreover, they use a form of direct communication to share important perceptual details that help them to complement their partial knowledge of the object shape. A leader robot manages the transport by compute the inter-robot distance and required bearing of each follower. The results show that the control architecture allow the group to transport an object along different predefined linear and curved trajectories known to the leader.

Finally, the main contribution of the work described in (Wan et al., 2017) is to test a caging strategy for transporting a triangular prism to a final destination by crossing a slope terrain. In this work, the control algorithm running on a master computer generates the minimum number of robots required to securely cage the object, the initial positions of each robot with respect to the object, and the robots motion during transport. The control method exploit a direct form of communication between the master computer and the robots, and makes use of a detailed knowledge of position and orientation of the robots and the object to be transported. The study shows that simulated robots can successfully transport objects of different shape and size along the slope terrain.

## Discussion

The research area targeting cooperative transport by MRSs is represented by an articulated and heterogeneous body of literature. We have chosen to illustrate this literature using a categorisation system that distinguishes the research works on the basis of the type of strategy used to collectively transport the object. In this section, we illustrate general patterns that emerge from the considerable methodological diversity illustrated in previous sections, and we identify open challenges and promising directions for further work.

Our review shows that, regardless of the type of transport strategy used, a certain amount of functional diversity among the members of a group seems to be an ineluctable methodological feature to allow the robotic systems to operate in an environment with obstacles, or to develop transport trajectories that adapt to varying environmental conditions. A robot leader, or a robot watcher as in (Gerkey and Matarić, 2002), is generally deputed to direct the transport by coordinating the actions and contributions of the followers. This is a particularly recurrent pattern in those research works based on the use of grasping strategies, where the fact of having all robots attached to the object facilitates the indirect communication and allocation of duties by the leader to the followers through force sensing mechanisms. In various studies exploiting the leader-follower approach, cooperative transport is exploited to cope with objects that due to their size can be hardly transported by a single robot. However, the transport strategies generated by these heterogeneous groups tend to be very fragile with respect to the object mass. This is because the object has to be light enough to respond to the forces exerted by the leader, that is often the only robot deputed to initiate the transport. The robustness with respect to the object mass tends to be more easily achieved by transport strategies developed by groups in which multiple agents can contribute to initiate and to sustain the transport. Another feature that tends to improve the robustness of the collective transport strategies with respect to the object mass, is the possibility for the robots to push each other as in (Fujisawa et al., 2013; Alkilabi et al., 2017), or to push and pull each other as in (Gross and Dorigo, 2009). In most of the studies we have reviewed in previous sections the robot-to-robot interactions are excluded by the use of control mechanisms designed to avoid robot-to-robot collisions. However, the exploitation of both the robot-to-robot and the robot-to-object interactions can facilitate the initial coordination of actions. Moreover, robot-to-robot interactions are particularly helpful in case of transport of heavy and relatively small objects, where the limited object perimeter prevents the group from developing the robot-to-object interactions required to initiate and sustain the transport.

When the heterogeneity of the system is the distinctive methodological feature that makes the group capable of operating in complex environments (e.g., environments with obstacles), it would be desirable if the allocation of roles or the emergence of any hierarchical organisation could be directly handled by the robots in an autonomous way. That would make possible the re-allocation of roles or the re-organisation of the group structure in case of failure of the *key element*. To the best of our knowledge, apart from the work described in (Gerkey and Matarić, 2002), heterogeneity is either based on structural differences among the robots, or on a type of functional differentiation a priori managed by the system’s designer. This means that the fault-tolerance is often partially or totally sacrificed in order to boost the competencies of the group. For the future, it would be interesting to see more research works focusing on the challenge of designing mechanisms to allow a group of robots to handle functional heterogeneity by allocating, and if necessary re-allocating, important functions in a completely autonomous way. That would reconcile fault-tolerance and group competencies to design MRSs capable of carrying out complex cooperative transport tasks.

In section 3, we have intentionally included in the category of grasping strategies those research works in which MRSs transport objects on top of their bodies, even if the robots do not use any special device to attach to the object. The logic behind this choice is that for both strategies (i.e., grasping and carrying the object on top of the body) the robots align their forces and sustain the transport without losing physical contact with the object. While for robots that carry the object on top of their bodies, the persistence of the physical contact with the object during the entire transport is generally an unavoidable consequence of the way in which the robots are meant to operate (these robots generally lack any grasping device), for robots that can grasp the object with a dedicated grasping device, the physical connection could in principle be released in particular during the initial phases of the transport to facilitate the alignment of pushing/pulling forces. To the best of our knowledge, the large majority of research works where MRSs use grasping strategies to collectively transport objects concern robots that are pre-attached to the object, and that never release the grasp during the transport (Tuci et al., 2006; Groß and Dorigo, 2008; Gross and Dorigo, 2009). The pre-attachment condition certainly takes out a large amount of complexity from research studies that tend to focus on the coordination of actions during the transport rather than on the distribution and alignment of pushing/pulling forces to initiate it. However, for the future, the development of mechanisms to allow robots to exploit both the grasp and the release action would be important not only to automate the distribution and the alignment of pushing/pulling forces, but also to improve the robustness of the system to be able to transport object of different shapes. We have seen that, apart from few studies, the large majority of the research works focus on the collective transport of rectangular objects (see Tables 1–3). The robustness of the collective transport strategies with respect to the object shape has been so far a rather neglected subject, that could be further investigated by researching and improving those aspects that, like the release and grasping process, directly affects it.

**Table 3:** Main characteristics of research works using a caging transport strategy.

	Experimental setting						Control and communication		Similar studies
	nr. robots	group type: -homogeneous (ho) -heterogeneous (he)	autonomous allocation of roles	env. with obstacles	obj. shape	transport direction	controller design method	communication	
Wang et al., 2004b	3	he	no	no	rectangular prism	known to leader	other	indirect	Wang et al., 2003; Wang et al., 2004a
Brown and Jennings, 1995	2	he	no	no	rectangular prism	known to leader	other	indirect	
Spletzer et al., 2001	3	he	no	no	rectangular prism	known to leader	other	indirect	
Pereira et al., 2004	3–4	ho	no	no	triangular prism	towards a goal known to all robots	other	direct	Wan et al., 2012
Fink et al., 2007	4–8	ho	no	yes	L-shaped	towards a goal known to all robots	behaviour-based	indirect	Fink et al., 2008; Eoh et al., 2014
Dai et al., 2016	3	he	no	no	convex polygon	predefined trajectories known to the leader	other	direct	
Wan et al., 2017	3	ho	no	no	triangular prism	predefined trajectories	other	direct	

See also caption Table 1 for further illustration of the descriptive categories.

Methodological alternatives to develop effective transport strategies for homogeneous groups required to operate in complex environments are generally limited to solutions that work only if the object to be transported does not occlude the robots’ view of the goal destination, or of the perception of eventual obstacles. The occlusion-based approach reviewed in section 2 (see Jianing Chen et al., 2015) discusses an algorithm that definitely overcomes the above-mentioned limitations and provides a very effective solution to allow homogeneous groups to cooperatively transport objects in an environment with obstacles. However, we point to the fact that, to the best of our knowledge, in the large majority of the reviewed studies, the initial alignment and the following coordination of actions is subject to the perception or to the occlusion (by the object to be transported) of the final destination of the transport (see Jianing Chen et al., 2015). This important assumption tends to simplify the initial process of the alignment of the transport forces, and largely undermines the robustness of the resulting group transport strategies to environment in which this assumption does not hold. We believe that the above mentioned assumption should be dropped to favour the robustness of the cooperative transport strategies. Finally, it is worth to note that no research work has been dedicated to the development of MRSs that can dynamically adjust the type of transport strategy (e.g., pushing, grasping, or caging) with respect to the characteristics of the object to be transported and/or of the environment in which the collective transport takes place. This is also a very interesting subject for future work.

## Conclusions

We have reviewed the literature on MRSs focused on the development of hardware and control systems to allow autonomous robots to cooperatively transport objects that can not be moved by a single robot. We have structured our review on a rather unconventional and relatively “coarse-grained” categorisation framework based on the type of transport strategy used by the robotic systems to move the objects. With this framework, we have ordered a rather heterogeneous body of MRSs literature, by focusing not only on motivations and objectives, but also on those distinctive methodological details that characterise the contribution of each single reviewed study. In section 5, we have critically examined common features emerging from the comparison of within and between categories works, and we have pointed to potentially fruitful directions for future work.

We wish to conclude this review with a brief reference to cooperative transport in natural systems. Ants have evolved extremely effective competencies to cooperatively retrieve items that can be hundreds or even thousands times the weight an individual can carry (Czaczkes et al., 2011). Owing to cooperative transport, ants can perform faster prey retrieval reducing both the exposition of foragers to predators, and the risk of food being caught and eaten by other aggressive species (Hölldobler et al., 1978; Yamamoto et al., 2009). The speedy retrieval of prey also reduces the time workers are involved in transport tasks, freeing them for other colony relevant tasks (Feener and Moss, 1990; Tanner, 2008). Cooperative transport also reduces the energy cost of transport by allowing carriers to keep up with the dense flow of traffic and by reducing the possibility of traffic jams (Czaczkes and Ratnieks, 2013). Biologists suggest that these complex group level responses are underpinned by simple behavioural rules (Franks, 1986; McCreery et al., 2016). We think that important lessons can still be learned from observing the complex cooperative transport behaviour shown by various ant species. It is then the task of roboticists to transform these observations into fruitful design principles and effective methodological choices to develop robust, flexible and scalable MRSs that cooperatively transport objects.

## Author Contributions

The authors have equally contributed to the analysis of the literature and to the writing of the paper.

## Conflict of Interest Statement

The authors declare that the research was conducted in the absence of any commercial or financial relationships that could be construed as a potential conflict of interest.
